# Striking errors in the methodology, execution, and conclusions of the Cochrane Library review of spinal cord stimulation for low back pain by Traeger *et al.*

**DOI:** 10.1093/pm/pnad047

**Published:** 2023-04-17

**Authors:** Shravani Durbhakula, Mustafa Y Broachwala, Nathaniel M Schuster, Zachary L McCormick

**Affiliations:** Department of Anesthesiology & Critical Care Medicine, Johns Hopkins School of Medicine, Baltimore, MD 21287, United States; Department of Physical Medicine & Rehabilitation, Johns Hopkins School of Medicine, Baltimore, MD 21287, United States; Department of Anesthesiology, University of California San Diego Health, San Diego, CA 92037, United States; Department of Physical Medical & Rehabilitation, University of Utah School of Medicine, Salt Lake City, UT 84132, United States

We write to the *Pain Medicine* community regarding the recent Cochrane review entitled “Spinal cord stimulation for low back pain” by Traeger et al.,^1^ as there is no direct forum for response to published Cochrane reviews. The review contains significant methodological issues. The authors then extrapolate questionable findings to generate overreaching conclusions that have the potential to negatively impact the care of patients with chronic low back pain (CLBP). Traeger et al.^1^ conclude that spinal cord stimulation (SCS) “probably has little to no sustained benefit over placebo for people with low back pain” and that the published data “do not support the use of SCS for people with low back pain outside a randomized, placebo-controlled trial.”^1^

When making broad recommendations about the use of SCS in the real world, the full context and breadth of available literature must be taken into consideration. The authors designed search criteria that included clinical trials comparing SCS to placebo or “no treatment” (including conventional medical management (CMM) studies with parallel-group design).^1^ However, they excluded large, multicenter comparative effectiveness trials and pragmatic studies—such as those comparing SCS against revision decompression and/or fusion surgery, and tonic SCS against novel waveforms.^1^ While we agree that placebo and sham controlled trials represent the highest level of scientific evidence, paresthesia-free waveforms which enable randomized, double-blind studies were developed merely over a decade ago. Furthermore, independent physician investigators have struggled to complete these studies as they are expensive to perform and recruitment is difficult for sham-controlled SCS trials. Meanwhile, industry is disincentivized from performing such studies, as the US Food and Drug Administration does not require them for regulatory approvals in the presence of a predicate device, they are costly and hard to accrue patients into, and the risk-benefit ratio does not favor companies and their shareholders. These historical limitations explain both the dearth of literature in this category and why the few published sham-controlled studies are smaller, single-center studies.

While high-quality placebo-controlled studies of SCS for CLBP are indeed needed, there is an abundance of Level 1 comparative effectiveness data that supports the effectiveness of SCS for CLBP. These studies have long-term follow-up and answer key clinical questions, such as defining the optimal SCS waveform for a specific patient phenotype, and whether SCS provides outcome and cost benefits over revision decompression and/or fusion surgery. The summation of these data demonstrates large magnitudes of effect, although with indirectness and potential for risk of bias. As such, GRADE assessment should reveal moderate-certainty evidence of medium to long-term effectiveness of SCS for CLBP.

Traeger et al.^1^ identified parallel trials evaluating SCS and conventional medical management (CMM) against CMM alone^1^; however, their interpretation of these studies and handling of inclusion/exclusion were misguided. Three parallel trials with medium-term follow up were originally included in a sub-analysis: Kapural et al.,^2^ Kumar et al.^3^ and Rigoard et al.^4^ Here, the authors grouped apples and oranges. Kumar et al.^3^ and Rigoard et al.^4^ both used older, conventional stimulation waveforms which are mechanistically distinct and less effective than the high frequency (10-kHz) stimulation^5^ used in Kapural et al.^2^ In addition to grouping different treatments, they combined outcome results relevant to different body regions. Unlike the other two studies, Kumar et al.^3^ included patients with primarily leg pain relative to CLBP (importantly, the predominance of CLBP was a main reason for exclusion) with the primary outcome being 50% reduction of leg pain. Regardless, Traeger et al.^1^ performed an aggregate analysis of all 3 studies. Participants who received SCS were 7.4 times more likely to report a 50% or greater improvement in pain compared to CMM alone.^1^ The authors then removed the Kapural et al.^2^ study from secondary analysis, dropping the estimated risk ratio to 4.2.^1^ They justified this by citing heterogeneity and too large of an effect size, based on I^2^ statistical analysis^1^—an analysis that is useful in large meta-analyses but inappropriate when assessing only three studies. Furthermore, if the Kumar et al.^3^ study had been removed originally as a study of leg pain, only Rigoard et al.^4^ and Kapural et al.^2^ would have remained in the sub-analysis. The elimination of an outlier when there are only two studies is nonsensical and raises serious concerns about Traeger et al.^1^ selectively “cherry-picking” studies to fit an agenda previously reflected by the same authors in a 2020 letter to the editor.^6^

The authors’ conclusions about SCS’s probable lack of efficacy rested singularly on the Hara et al.^7^ study, a placebo-controlled trial with medium-term follow up. Hara et al.^7^ was published on October 18, 2022 despite the authors’ original search including ongoing trials up to June 10, 2022 only. Instead of repeating the full search in October and including all new evidence, the authors manually included Hara et al.^7^ post hoc.^1^ This methodological misadventure is puzzling, as the Traeger et al.^1^ author group previously criticized a prior author group for poor “conduct in systematic reviews”^6^ that could lead to “misleading” conclusions.^6^ Moreover, Traeger et al.^1^ curiously assigned “moderate-certainty”^1^ evidence to the statement that SCS “probably has little to no sustained benefit over placebo for people with low back pain,”^1^ dismissing the many flaws of the Hara et al.^7^ study. Such flaws have been described by expert pain physicians and clinical scientists in the neuromodulation field from various parts of the world. Multiple responses in JAMA^8^^,^^9^ and other medical journals^10^ outline the lack of validity of the Hara et al.^7^ study based on: 1) trialing with tonic stimulation rather than the experimental burst SCS waveform used at implant, 2) allowing placebo-level responders to pass into the implant phase, 3) using a single, ineffective waveform which is not used as monotherapy in clinical practice (40-Hz burst mode of constant current stimuli with 4 spikes per burst and an amplitude corresponding to 50%–70% of the paresthesia perception threshold), effectively rendering it a placebo versus placebo trial, and 4) trialing followed by randomization after the trial, which is inconsistent with other SCS studies and masks the true high attrition rate (65 trialed, 42 completed all randomization periods and had ODI measurements at all follow-up visits^7^) As such, even with its inclusion, an informed interpretation of the Hara et al.^7^ study results in a global assessment that the moderate to long-term efficacy of SCS compared to placebo is “inconclusive”, rather than “probably” providing “no sustained benefit.”.^1^

Generalizations about CLBP care interventions, drawn from data limited by narrow search criteria, are problematic and misleading. The reader is left questioning whether Traeger et al.^1^ intended to provide a balanced assessment of the published literature on SCS for CLBP from conception, given their 2020 letter to the editor^6^ and stated conflicts of interest that include royalties from two books: 1) *Surgery, the ultimate placebo* and 2) *Hippocrasy: How doctors are betraying their oath.* Furthermore, while a pain physician was acknowledged at the end of the publication,^1^ the extent of this physician’s involvement was too limited to warrant authorship. Inclusion of a physician author with content expertise in pain medicine and neuromodulation could have offered insight into the limitations addressed here, provided a balanced interpretation of the published literature, and aided with recommendations of appropriate scope.

We respectfully urge the Cochrane Library to retract and revise the Traeger et al.^1^ study or, at minimum, publish a corrigendum addressing our concerns. An appropriate revision should include, 1) re-evaluation of the search methodology to ensure a comprehensive selection of studies, 2) appropriate interpretation and synthesis of studies based on their inclusion/exclusion criteria allowing for valid findings, 3) diversification of authorship to include contributions from individuals with clinical and content expertise in neuromodulation for CLBP, and 4) conclusions of appropriate scope. By doing this, the Cochrane Library can contribute to a more accurate and balanced understanding of SCS for CLBP, ultimately benefiting patients, clinicians, researchers, payors, and policy makers.
